# Exosomes secreted by urine-derived stem cells improve stress urinary incontinence by promoting repair of pubococcygeus muscle injury in rats

**DOI:** 10.1186/s13287-019-1182-4

**Published:** 2019-03-08

**Authors:** Ruoyu Wu, Chengsheng Huang, Qingkai Wu, Xiang Jia, Mengyu Liu, Zhuowei Xue, Yu Qiu, Xin Niu, Yang Wang

**Affiliations:** 1 Department of Obstetrics and Gynecology, Shanghai Jiao Tong University Affiliated Sixth People’s Hospital, Shanghai, 200233 People’s Republic of China; 2 Institute of Microsurgery on Extremities, Shanghai Jiao Tong University Affiliated Sixth People’s Hospital, Shanghai, 200233 People’s Republic of China

**Keywords:** Exosomes, Stem cell, Stress urinary incontinence

## Abstract

**Background:**

Previous studies revealed that urine-derived stem cells (USCs) could promote myogenesis after the impairment of the sphincter muscles. However, the effects of exosomes secreted by USCs (USCs-Exo) were not elucidated. Exosomes are nanosized membrane vesicles secreted by the cells. They have been proved to be effective in protecting against tissue injury and therapeutic in tissue repair. USCs are ideal sources of exosomes because of the noninvasive obtaining method and self-renewal abilitiy. This study aimed to show the therapeutic effects of USCs-Exo on improving stress urinary incontinence (SUI).

**Methods:**

Rat SUI models were established in this study using vaginal balloon inflation, and urodynamic and histological examination were carried out after exosome application. The proliferation and differentiation of muscle satellite cells (SCs) were evaluated using EdU, Cell Counting Kit 8, immunofluorescence staining, and Western blot analysis. mRNAs and proteins related to the activation of SCs were detected by reverse transcriptase quantitative polymerase chain reaction (RT-qPCR) and Western blot analysis.

**Results:**

After exosome injection, the urodynamic parameters significantly improved and the injured muscle tissue recovered well. The activation, proliferation, and differentiation of SCs were promoted. The phosphorylation of extracellular-regulated protein kinases (ERK) was enhanced. When ERK was inhibited, the promoting effect of USCs-Exo treatment disappeared.

**Conclusion:**

The findings of this study elucidated the functional roles of USCs-Exo in satellite cell ERK phosphorylation and identified a novel agent for skeletal muscle regeneration, providing a basis for further exploring a cell-free correction for SUI.

**Electronic supplementary material:**

The online version of this article (10.1186/s13287-019-1182-4) contains supplementary material, which is available to authorized users.

## Background

Stress urinary incontinence (SUI) is usually caused by pelvic floor muscle injury (mainly the pubococcygeus muscle) and affects up to 50% of adult women [1, 2]. At present, the Kegel exercise is widely used to help restore the contractility of the pubococcygeus muscle and improve SUI. Previous studies have also revealed effects of stem cells in correcting SUI [3]. Notably, several studies indicated that the therapeutic effects of stem cells might not be entirely exerted by the stem cells themselves. Stem cell paracrine action might also be a potential mechanism [4–6].

Exosomes are vesicles (diameter 30–150 nm) secreted by various cells. They traffic proteins, cytokines, and mRNAs between the cells [4, 7–9] and have been proved to be effective in protecting against tissue injury and therapeutic in tissue repair [10–13]. Exosomes secreted by stem cells have been shown to function like stem cells but avoid the potential risks during cell transplantation [14]. Exosomes have been proved to be useful in accelerating skeletal muscle regeneration [15]. Urine-derived stem cells (USCs) can be obtained by noninvasive methods and are easy to culture and proliferate, which makes them an ideal source for exosome extraction [16, 17]. Our previous studies demonstrated that exosomes secreted by urine-derived stem cells (USCs-Exo) could promote wound healing and bone regeneration [18, 19]. USCs-Exo also have the properties of promoting angiogenesis, repairing lower limb ischemia [20], and preventing diabetic nephropathy [21]. Therefore, paracrine USCs-Exo might be an advantageous alternative to stem cells and important for correcting SUI. This study elucidated the functional role of USCs-Exo in correcting SUI. USCs-Exo could enhance the phosphorylation of ERK and promote the activation, proliferation, and differentiation of satellite cells (SCs), thereby promoting the recovery of the damaged pubococcygeus muscle and improving the symptoms of SUI.

## Methods

### Isolation and identification of USCs

Human USCs were isolated from healthy young women and identified as previously reported [18, 22]. Briefly, a total of 10 fresh voided urine samples (200 mL each) supplemented with 1% penicillin and 1% streptomycin (Gibco, USA) were collected. After centrifugation at 400 × *g* for 10 min at room temperature, the supernatant was discarded, and the sediment was washed twice with phosphate-buffered saline (PBS; Hyclone, USA). Then, the sediment was re-suspended in Dulbecco’s modified Eagle medium (DMEM; Hyclone) supplemented with 2% (*v*/*v*) fetal bovine serum (FBS; Gibco), 10 ng/mL human epidermal growth factor (hEGF), 2 ng/mL platelet-derived growth factor (PDGF), 1 ng/mL transforming growth factor (TGF-β), 2 ng/mL basic fibroblast growth factor (bFGF), 0.5 μM hydrocortisone, 25 μg/mL insulin, 20 μg/mL transferrin, 549 ng/mL epinephrine, 50 ng/mL triiodothyronine (T3), l-glu, and antibiotics. Cell suspension was plated in gelatin-coated 24-well plates (Corning, USA) and incubated in a humidified atmosphere with 5% CO_2_ at 37 °C. After 7 days, the medium was replaced. Adherent colonies derived from single cells were marked and used as independent clones while non-adherent cells were washed away. The culture medium was refreshed every other 3 days. When the cells reached approximately 80% confluence, they were passaged using 0.25% trypsin (Additional file 1).

The surface antigens of USCs were analyzed by flow cytometry (FCM). The cells at passage 3 were incubated with 3% bovine serum albumin (BSA; Gibco) in PBS for 30 min to block non-specific antigens. Then, the cells were incubated with following antibodies (Becton-Dickinson, USA) against the USC surface markers: CD29, CD34, CD45, CD73, CD90, and human leukocyte antigen DR (HLA-DR). The cells were washed three times to remove unbound antibodies and then incubated with second antibodies for 30 min. A flow cytometer (Guava easyCyteTM; Milipore, USA) was used to analyze the surface antigens.

### Isolation and identification of USCs-Exo

USCs-Exo were prepared and purified as previously reported [23, 24]. Eighty to ninety percent confluence USCs were washed with PBS and then cultured in serum-free conditioned medium (CM) for another 48 h. CM was harvested and centrifuged at 300 × *g* for 10 min, then at 2000 × *g* for 10 min at 4 °C (Beckman, CA, USA). CM was filtered using a 0.22-μm syringe filter unit (Millipore, USA) after centrifugation to exclude the cellular debris. The supernatants were ultra-centrifuged at 100,000 × *g* for 2 h at 4 °C to precipitate exosome pellets. The pellets were suspended in PBS and centrifuged at 4000 × *g* for 10 min. Finally, the exosome pellets were re-suspended in 200 μL of PBS. USCs-Exo fraction was assessed by a Hitachi H-7650 transmission electron microscope (TEM; Hitachi, Japan). The exosome pellets were loaded to copper grids coated with formvar after fixed in 3% (*w*/*v*) glutaraldehyde and 2% paraformaldehyde in cacodylate buffer. After washing and evaporation, the grids were contrasted in 2% uranyl acetate and examined by TEM. The identity of USCs-Exo was confirmed by the presence of the specific surface proteins: CD9, CD63, CD81, and TSG101 [25, 26]. The operations of Western blot would be detailed at the end of this section. Assessments of size distribution and concentration of USCs-Exo were implemented by tunable resistive pulse sensing (TRPS) analysis which is a qNano platform with an NP100-rated nanopore (Izon Science, UK). CPC100 particles (Izon Science) were used to calibrate the size and concentration following manufacturer’s instructions, and the membrane was stretched at 47.0 mm. USCs-Exo sample was diluted 50-fold with 0.22-μm filtered PBS and measured for 5 min. Izon Control Suite software v2.2 (Izon Science) was used for data processing and analysis.

### SUI rat model and injection of USCs-Exo

All procedures were approved by the Animal Research Committee at Shanghai Sixth People’s Hospital and carried out in accordance with the approved guidelines, laboratory animal license number SYXK(Hu)-2016-0020. The method for establishing a rat SUI model refers to the study of Lin et al. [27]. Female Sprague-Dawley rats (8 weeks of age) were anesthetized with ketamine (50 mg/kg) intraperitoneally, and the forelimbs were fixed and dragged on the cage with a supine position. A catheter was inserted 2~3 cm into the rat’s vagina and fixed with a single 3-0 surgical needle. Then, the balloon was inflated with 5-mL sterile saline. The catheter was suspended from cage without contacting the tabletop. At the end of catheter, there was a water bag weighing about 0.3 kg. As described above, a traction parallel to the vagina was given for 8 h. One week after injury, maximum bladder volume (MBV) and abdominal leak point pressure (ALPP) were tested to decide whether the SUI model was created. The operations were detailed in Additional file 2. Then, 40 SUI rats were randomly divided into two groups and 20 rats in each group (for each group *n* = 20). One group was given USCs-Exo 1 × 10^10^ particles/mL 1 mL local injection in and around the pubococcygeus muscle. The other group was given 1 mL 0.9% physiological saline local injection in and around the pubococcygeus muscle. Five rats of both groups were tested for urodynamic parameters (MBV, ALPP) before injection (0 week) and at 2, 4, and 8 weeks after injection (for each time point *n* = 5).

### Histological and immunohistochemistry analysis

Rats were sacrificed under anesthesia after ALPP and MBV measurement. The proximal pubococcygeus was removed by an expert and fixed in 10% formalin for hematoxylin and eosin (H&E) staining, Masson’s trichrome staining, and immunohistochemistry staining. Tissue specimens were embedded in paraffin and sliced into 4-μm-thick sections. H&E staining was used for histological observations, while Masson’s trichrome staining determined the degree of collagen maturity. Immunohistochemistry was used for determining striated muscle area in each section. Rat MHC antibody was utilized. The quantitative morphometry of pubococcygeus was conducted using a light microscope at × 10 magnification (DM 4000B, Leica Micro systems, Buffalo Grove, IL, USA). For the evaluation of nuclear ingression, five randomly selected areas at × 100 magnification were evaluated. Nuclear ingression was recognized as nucleus located in the center of the newborn muscle fibers. Fibrosis ratio was analyzed by software Image-Pro Plus 6.0.

### SC quiescency, activation, and cell cycle changes

Passage 4 cells were seeded into a six-well plate at 100,000 cells/well. After adherence, the culture medium was discarded and cells were starved with DMEM only, for 24 h. Then, the medium was changed and added with USCs-Exo at 1 × 10^10^ particles/mL. Immunofluorescent staining was exerted to detect Pax7 and MyoD at 8, 16, 24, and 32 h.

Another sample was prepared for RT-qPCR. The cells were cultured with normal medium, and USCs-Exo were given at 1 × 10^10^ particles/mL. After 12 h, the cells were harvested for RT-qPCR. There are none-USCs-Exo-treated SCs taken as control. The operation of immunofluorescent staining and RT-qPCR would be detailed at the end of this section.

### Promotion of ERK phosphorylation and change of mRNAs downstream Ras-ERK signaling pathway

Passage 4 cells were seeded into a six-well plate at 100,000 cells/well. The medium was added with USCs-Exo at 1 × 10^10^ particles/mL. After 12 h, the cells were harvested for Western blot for ERK phosphorylation. RT-qPCR was also carried out for examination of Ras-ERK signaling pathway and mRNAs downstream. No USCs-Exo-treated SCs were taken as control. The operation of Western blot and RT-qPCR would be detailed at the end of this section.

### Inhibitor and antibodies

SCs were cultivated with DMEM (10%FBS, 1% penicillin, and streptomycin). In USCs-Exo group, USCs-Exo were added into culture medium at 1 × 10^10^ particles/mL. PD98059 (Selleck Chemicals, USA) was used as ERK phosphorylation inhibitor. In PD98059 group, the inhibitor was dissolved in DMSO and then added into the culture medium at the concentration of 150 μM. In USCs-Exo + PD98059 group, both USCs-Exo and inhibitor mentioned above were added into the culture medium. The cells were incubated for another 24 h and then examined for proliferation and differentiation. USCs-Exo and normal cultured (control) groups and USCs-Exo, USCs-Exo + PD98059, and PD98059 groups were examined separately.

Antibodies involved are listed in Additional file 2.

### SC proliferation assays

Passage 4 cells were seeded into a six-well plate at 50,000 cells/well. When the cells reached 50% confluence, the medium changed as described above. After 24 h, cell proliferation was tested by a EdU-Click 488 proliferation kit (Sigma-Aldrich, USA) according to the manufacturer’s instructions. Samples were observed with a fluorescence microscope (DMI 6000, Leica Micro systems, Buffalo Grove, IL, USA).

Passage 4 cells were seeded into a 96-well plate at 1000 cells/well. After cell adherence, the medium was changed as described above. According to the manufacturer’s instructions, cell proliferation was assayed on days 1, 2, 3, 4, 5, 6, and 7. Ten microliters of the reagent with Cell Counting Kit-8 (CCK-8; Dojindo, Japan) was added to each well, and the plates were incubated at 37 °C for 2–4 h. Finally, the absorbance was measured at 450 nm using a Microplate Reader (iMarkTM; Bio-Rad, USA).

### Myogenic differentiation of SCs

Passage 4 cells were seeded into six-well plates at 50,000 cells/well. When the cells reached 90% confluence, the medium changed as described above. After 24 h, the cells were observed after immunofluorescence staining. The expression of myogenic proteins was assessed by Western blot. The operations of immunofluorescence staining and Western blot would be described at the end of this section.

### RNA isolation and expression analysis

The total RNA of samples was extracted using Trizol-up (EZBioscience). An EZBioscience 4× Reverse Transcription Master Mix (EZBioscience) was used for reverse transcription reaction. The EZBioscience 2× SYBR Green qPCR Master Mix (EZBioscience) was used to perform RT-qPCR. Subsequent real-time PCR analysis was carried out using an ABI 7900 HT Sequence Detection System. The cycling steps were as follows: reverse transcription (30 min, 50 °C), denaturation (2 min, 95 °C), and 40 amplification cycles (15 s at 95 °C and 1 min at 60 °C). The mRNA levels were calculated by 2–ΔΔCt with GADPH as internal control. The primers were detailed in Additional file 2.

### Immunofluorescence staining

The cells were washed three times with preheated PBS (1×) and 10 min each time. Then, the cells were fixed with 4% formaldehyde at room temperature for 30 min. After that, the cells were permeabilized with 0.2% Triton X-100 for 5 min and blocked with 5% goat serum at room temperature for 30 min. Then, the cells were incubated with primary antibodies (diluted with 1% goat serum) overnight, in a wet box at 4 °C. On the next day, the cells were incubated with secondary antibodies in the darkroom. Cells were counterstained with 1 μg/mL hoechst for 1 min and then observed with a fluorescence microscope (DMI 6000, Leica Micro systems, Buffalo Grove, IL, USA). Between operations, the cells were washed three times with preheated PBS (1×) and 10 min for each time. The antibodies were detailed in Additional file 2.

### Western blot

The proteins were extracted from the cells using a cell lysis buffer supplemented with proteinase inhibitor and then centrifuged at 14000 g for 15 min. A BCA protein assay kit (Beyotime, China) was used to measure the amount of protein. The samples were added with 5× protein-loading buffer (CST, USA) and denatured at 95 °C for 10 min. Then, protein was loaded into prepared 10% (*w*/*v*) SDS-PAGE gel (15% for those < 20 kD). The sample was run at 80 V for 90 min and transferred onto 0.22-μm polyvinylidene difluoride membranes (Millipore, USA) for 1.5 h at 100 mA (300 mA for those > 20 kD). Then, the membrane was blocked with 5% milk in Tris-buffered saline 0.1% Tween (TBST) for 1 h and incubated with primary rabbit polyclonal antibodies. Subsequently, the membranes were washed three times in 1× Tris-HCl-buffered saline with Tween 20 (TBS and 0.1% Tween 20; TBST) for 10 min and incubated for 1 h in with HRP-conjugated goat anti-rabbit secondary antibody (Abcam). The proteins were detected using enhanced chemiluminescence (Thermo Fisher, USA), and the immunoreactive bands imaged using an Image Quant LAS 4000 mini biomolecular imager (Bio-Rad, USA).

### Histological and statistical analysis

ImageJ (National Institutes of Health, USA) was used to analyze Western blot, immunofluorescent staining, and histological examination. GraphPad Prime 6 (GraphPad, USA) was used to analyze the data in all groups. The data were reported as mean ± standard deviation (SD). The comparisons between groups were carried out with Student’s *t* test and one-way ANOVA. Trend forecasts were carried with two-way repeated measures ANOVA. Statistical differences were considered as significant at *P* < 0.05.

## Results

### Characterization of USCs

**Fig. 1:**
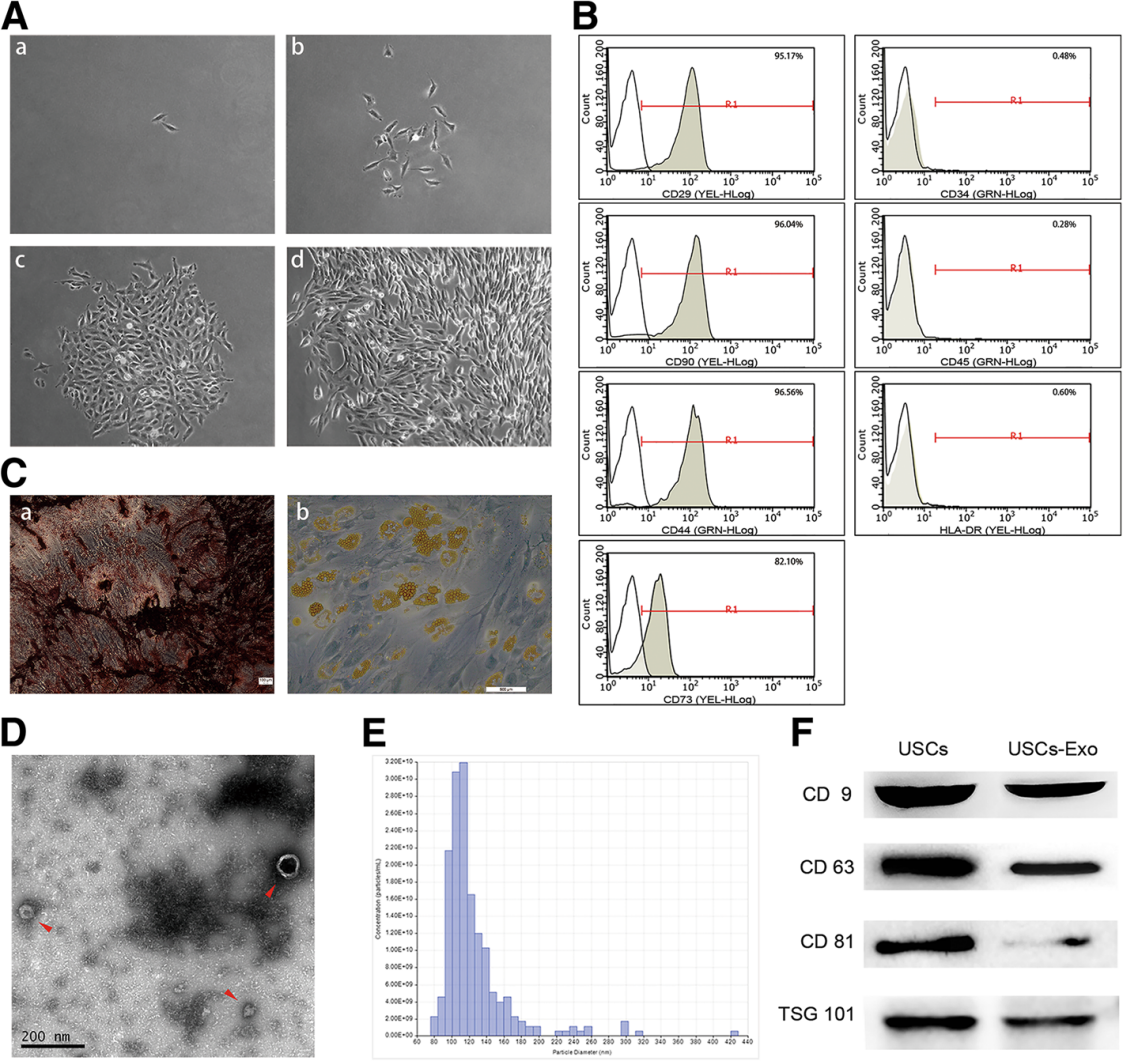
Characterization of USCs and USCs-Exo. **A** Morphology and growth of USCs. **B** USCs were characterized by FCM using the surface markers CD29, CD90, CD73, CD44, CD34, CD45, and HLA-DR. **C** USC osteogenic (**C-a**) and adipogenic (**C-b**) differentiation. **D** Morphology of USCs-Exo under a transmission electron microscope (arrowheads). **E** TRPS measurement showed that the size range of USCs-Exo concentrated at 70–150 nm, and the measured mean concentration (particles/mL) of USCs-Exo was 1.57 × 10^11^). **F** Western blot analysis of exosome-specific CD9, CD63, CD81, and TSG101 protein expressions in USCs and USCs-Exo

The cell colony was observed about 7 days after initial plating. USCs were confirmed with a fibroblast-like morphology using light microscopy (Fig. 1A). The cells achieved 80–90% confluence on day 12. After several passages, the fibroblast-like morphology was observed (Fig. 1A-d). FCM analysis demonstrated that USCs were positive for CD29, CD73, CD90, and CD44 antigen and negative for CD34, CD45, and HLA-DR (Fig. 1B). USCs can differentiate into osteoblasts (Fig. 1C-a) and adipose (Fig. 1C-b) cells, which is consistent with the existing research [18, 22]. The characterization of USCs meets the criteria for defining multipotent MSCs.

### Characterization of USCs-Exo

The particle size and concentration were measured by TRPS, and their morphology was observed using a TEM. The diameters of USCs-Exo were approximately 50–100 nm tested by TRPS analysis (Fig. 1D) and about 100 nm observed by TEM (Fig. 1E). Exosome markers, including CD9, CD63, CD81, and TSG101, were expressed in USCs-Exo, as shown by Western blot analysis (Fig. 1F).

### USCs-Exo improved the urodynamic parameters in rats with SUI

**Fig. 2:**
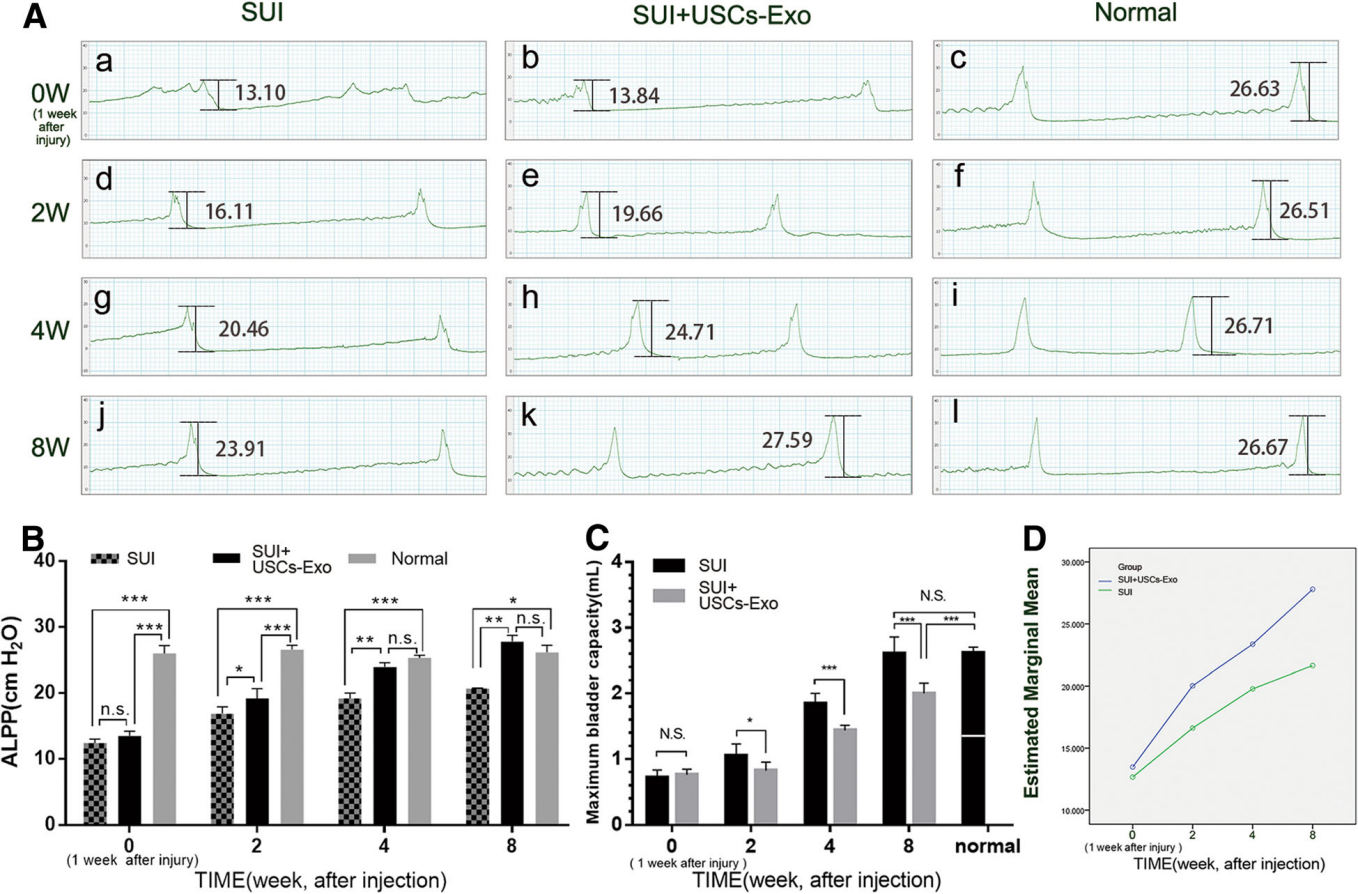
Improvements in the urodynamic parameters in rats with SUI. **A** Before treatment (1 week after injury) (**A-a**, **A-b**) ALPP of both SUI + USCs-Exo and SUI groups decreased significantly compared with normal group (**A-c**), 2 and 4 weeks after the injection of USCs-Exo, ALPP of SUI + USCs-Exo group increased greatly (**A-e**, **A-h**) compared with SUI group (**A-d**, **A-g**). Eight weeks after the injection, ALPP of SUI + USCs-Exo group reached a normal level (**A-k**). **B** The ALPP statistics displayed that the differences in ALPP in the SUI group between each time point were not significant, mostly. However, the situation was totally different in the SUI + USCs-Exo group and no difference was found in the ALPP compared with normal group in week 8. **C** Statistics of MBV showed that on injecting USCs-Exo, the MBV recovered gradually and reached a normal level, finally, while the SUI group did not. **D** Estimated marginal mean displayed whether injecting USCs-Exo led to different recovery trend and using USCs-Exo caused better recovery (*P < 0.05, **P < 0.01, ***P < 0.001; n.s., no significance)

Forty rats with a positive sneeze test and significant decreases in urodynamic parameters (abdominal leak point pressure, ALPP, maximum bladder volume, MBV) after 1 week of injury were included in the SUI model and investigated in this study. ALPP and MBV were measured in each group in weeks 2, 4, and 8 after USCs-Exo injection. In this study, ALPP and MBV in normal rats were 26.11 ± 0.88 cmH_2_O (Fig. 2A-c, A-f, A-i, A-l) and 2.62 ± 0.28 mL (Fig. 2C). After pubococcygeus muscle injury, the urodynamic parameters were significantly reduced (Fig. 2A-a, b; C). After USCs-Exo injection, ALPP and MBV of rats significantly improved. The SUI group without USCs-Exo injection did not show the same improvements (Table 1; Fig. 2A, B, C). The difference in ALPP and MBV between SUI + USCs-Exo and normal groups was not statistically significant in week 8 (Fig. 2B, C). This indicated that USCs-Exo could make the full recovery of ALPP and MBV in 8 weeks (or less time). This estimated marginal mean also displayed that the injection of USCs-Exo led to a better recovery trend compared with the control group (Fig. 2D).

**Table 1:** Changes of urodynamic parameters in SUI rats

	ALPP (cmH_2_O)		MBV (mL)	
	SUI	SUI + USCs-Exo	SUI	SUI + USCs-Exo
Week 2	16.62 ± 0.86	19.19 ± 1.48	0.76 ± 0.08	1.05 ± 0.18
Week 4	19.38 ± 0.81	23.91 ± 0.69	1.44 ± 0.08	1.85 ± 0.16
Week 8	21.64 ± 1.64	27.80 ± 0.94	1.99 ± 0.07	2.60 ± 0.26
Normal	26.11 ± 0.88		2.62 ± 0.28	

### USCs-Exo promoted the recovery of the pubococcygeus muscle

**Fig. 3:**
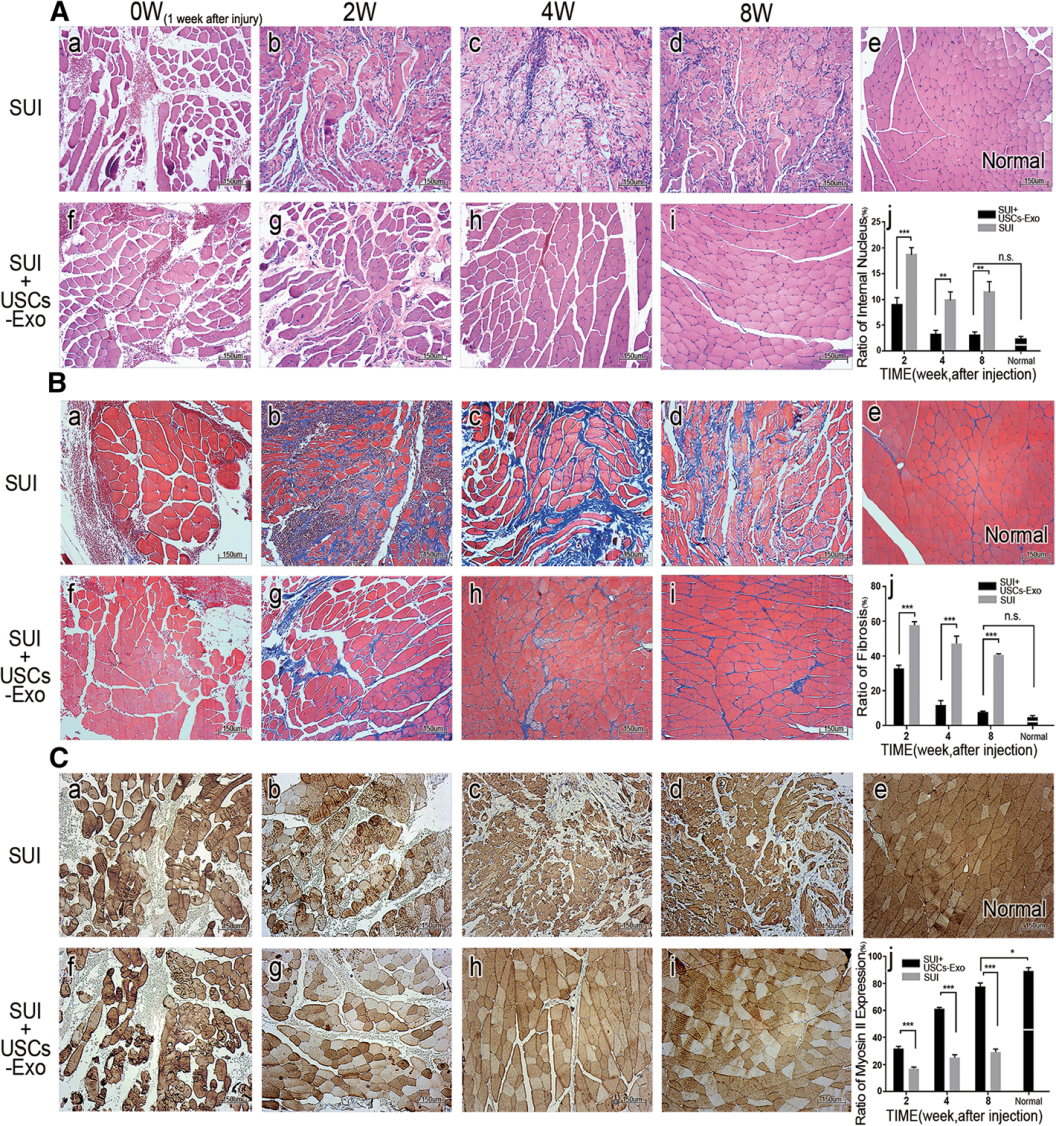
USCs-Exo promoted the recovery of the pubococcygeus muscle. **A** HE staining of the pubococcygeus muscle. In the SUI group, the newborn muscle fibers varied in size and shape and remained disorder (**A-b**, **A-c**, **A-d**). However, the muscle bundle structures in the SUI + USCs-Exo group (**A-g**, **A-h**, **A-i**) recovered to almost normal (**A-e**). Nuclear ingression was observed in both groups (**A-j**). **B** Masson staining of the pubococcygeus muscle: 8 weeks after the injection, fibrosis and muscle morphology were close to normal in the SUI + USCs-Exo group, while muscle atrophy and vicarious hypertrophy were still observed and the muscle structure was disordered in the SUI group. Fibrosis (**B-j**). **C** Immunohistochemistry analysis of the pubococcygeus muscle. Expression of myosin II (dark stained) was different in the two groups. Expression of myosin II was more in the SUI + USCs-Exo group than in the SUI group. Also, the expression of myosin II increased, gradually (**C-j**) (*P < 0.05, **P < 0.01, ***P < 0.001; n.s., no significance; × 100 magnification)

Impairment of the pubococcygeus muscle is usually an important cause of female SUI [28, 29]. The pubococcygeus muscle bundles broke, the size and shape of muscle fibers were irregular, blood vessels were destroyed, and hemocyte migration occurred 1 week after the injury (Fig. 3A-a, A-f). The SUI group showed rupture of muscle fibers accompanied by necrosis and atrophy. Newborn muscle fibers were morphologically irregular, and atrophy was observed accompanied by compensatory hypertrophy (Fig. 3A-b, A-c, and A-d). Inflammatory cell infiltration was also observed during the repair. USCs-Exo could prevent ruptured muscle fibers from secondary necrosis, maintain the shape of the muscle bundle, and promote normal muscle regeneration (Fig. 3A-g, A-h, and A-i). Also, a decreased nuclear ingression was observed in the SUI + USCs-Exo group (Fig. 3A-j). Masson staining showed that the morphology of the new muscle tissue was disordered in SUI group. Muscle fiber atrophy and compensatory hypertrophy were observed, together with increased fibrosis (Fig. 3B-b, B-c, and B-d). However, USCs-Exo could significantly reduce fibrosis and promote muscle morphological recovery (Fig. 3B-g, B-h, B-i). The fibrosis in the USCs-Exo group decreased and became close to normal (Fig. 3B-e, B-j). Immunohistochemical staining showed that the bundles of the new muscles were of different sizes and irregular shapes in the SUI group, and some of them had compensatory hypertrophy (Fig. 3C-b, C-c, and C-d). However, the muscles gradually recovered to normal in the SUI + USCs-Exo group (Fig. 3C-e). Myosin II are dark fibers in the figure which reflected muscle contraction indirectly. Obviously, the SUI + USCs-Exo group showed a progressive increase in the expression of myosin II, indicating that the muscle contraction ability also gradually recovered (Fig. 3C-g, C-h, and C-i). The increase in myosin II could also be evaluated, as shown in Fig. 3C-j.

### USCs-Exo could promote satellite cell activation

**Fig. 4:**
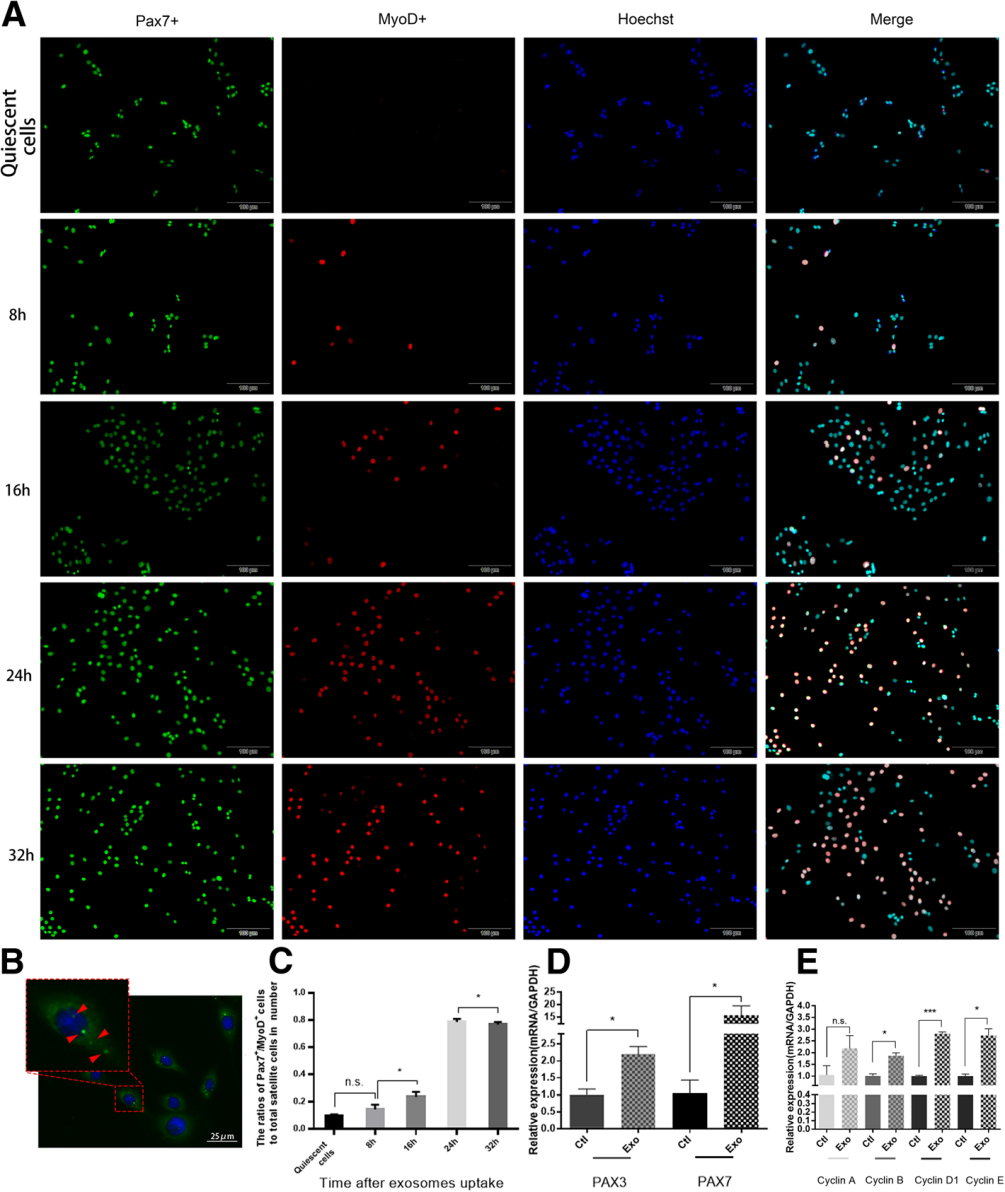
Exosome uptake and acceleration of the activation of SCs. **A** Pax7^+^ cells were stained green, while MyoD^+^ cells were stained red. Nuclei were stained blue. After cultivation with USCs-Exo, the number of activated SCs (Pax7^+^/MyoD^+^) increased significantly. **B** Exosome uptake. Exosomes were stained green and shown as bright green dots (arrowheads), while the nuclei were stained blue. **C** Statistical result of SC activation. The cells were activated after cultivation with USCs-Exo, and the number of activated cells reached a maximum at 24 h. **D** RT-PCR results of Pax3 and Pax7 mRNAs. The expression of Pax3 and Pax7 in SCs increased after cultivation with USCs-Exo. **E** RT-PCR results of cyclin mRNAs. The expression of cyclin B, cyclin D1, and cyclin E increased after cultivation with USCs-Exo (Exo, USCs-Exo; ctl, control; *P < 0.05, ***P < 0.001; n.s., no significance; × 100 magnification)

Activation of SCs is the first step of muscle regeneration. Therefore, the role of USCs-Exo in SC activation was investigated in this study. Immunofluorescence staining suggested that few SCs expressed MyoD after 24 h of inhibition (Fig. 4A). The Pax7+/MyoD+ rate of stem cells significantly increased and peaked (Fig. 4A, C) 24 h after USCs-Exo uptake (Fig. 4B). RT-qPCR was used to detect mRNAs related to SC activation. The expression of Pax3/7, cyclin B, cyclin D1, and cyclin E in SCs was upregulated to different degrees after USCs-Exo stimulation (Fig. 4D, E). The aforementioned results suggested that USCs-Exo could promote the activation of SCs.

### USCs-Exo promoted the proliferation and differentiation of SCs

**Fig. 5:**
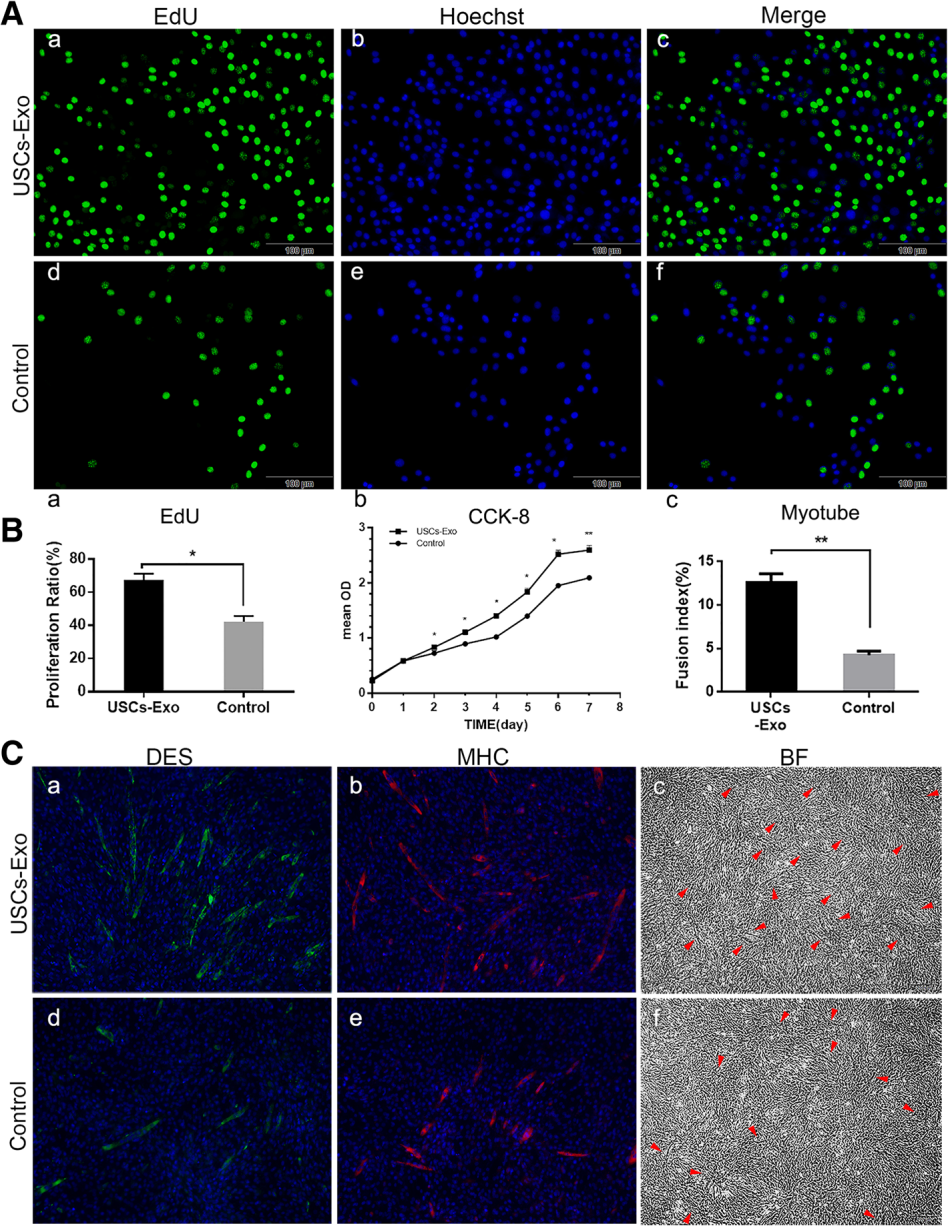
USCs-Exo promoted the proliferation and differentiation of SCs. **A** EdU staining after 24-h stimulation. Proliferative cells were stained green with Edu. All live cells were stained blue with hoechst. **B-a** Proliferation ratio of SCs in the EdU test. **B-b** CCK-8 test showed that USCs-Exo could promote the proliferation of SCs. **B-c** Myotube formation of SCs was shown as fusion index. **C** Twenty-four hour cultivation after USCs-Exo application. The myotube formation was promoted in the USCs-Exo group (**C-a**, **C-b**, **C-c**). DES was stained green and MHC was stained red. Nuclei were stained blue. Myotubes were observed as stained DES, MHC, and arrowheads in bright field (BF) (*P < 0.05, **P < 0.01; × 100 magnification)

Proliferation and differentiation of SCs under the stimulation of USCs-Exo were evaluated by the EdU test, CCK-8 test, and immunofluorescence staining. The EdU test showed that the activity of proliferation of SCs increased significantly after USCs-Exo stimulation (Fig. 5A, B-a). CCK-8 test showed that cell proliferation was obviously enhanced in the presence of USCs-Exo (Fig. 5B-b). Meanwhile, more myotubes formed in the USCs-Exo group than in the control group (Fig. 5B-c). Myotubes were observed as desmin (DES, stained as green tubes) and myosin heavy chain (MHC, stained as red tubes). They could also be observed in bright field (arrowheads; Fig. 5C-c, C-f).

### USCs-Exo promoted ERK phosphorylation and expression of mRNA downstream Ras-ERK signaling pathway

**Fig. 6:**
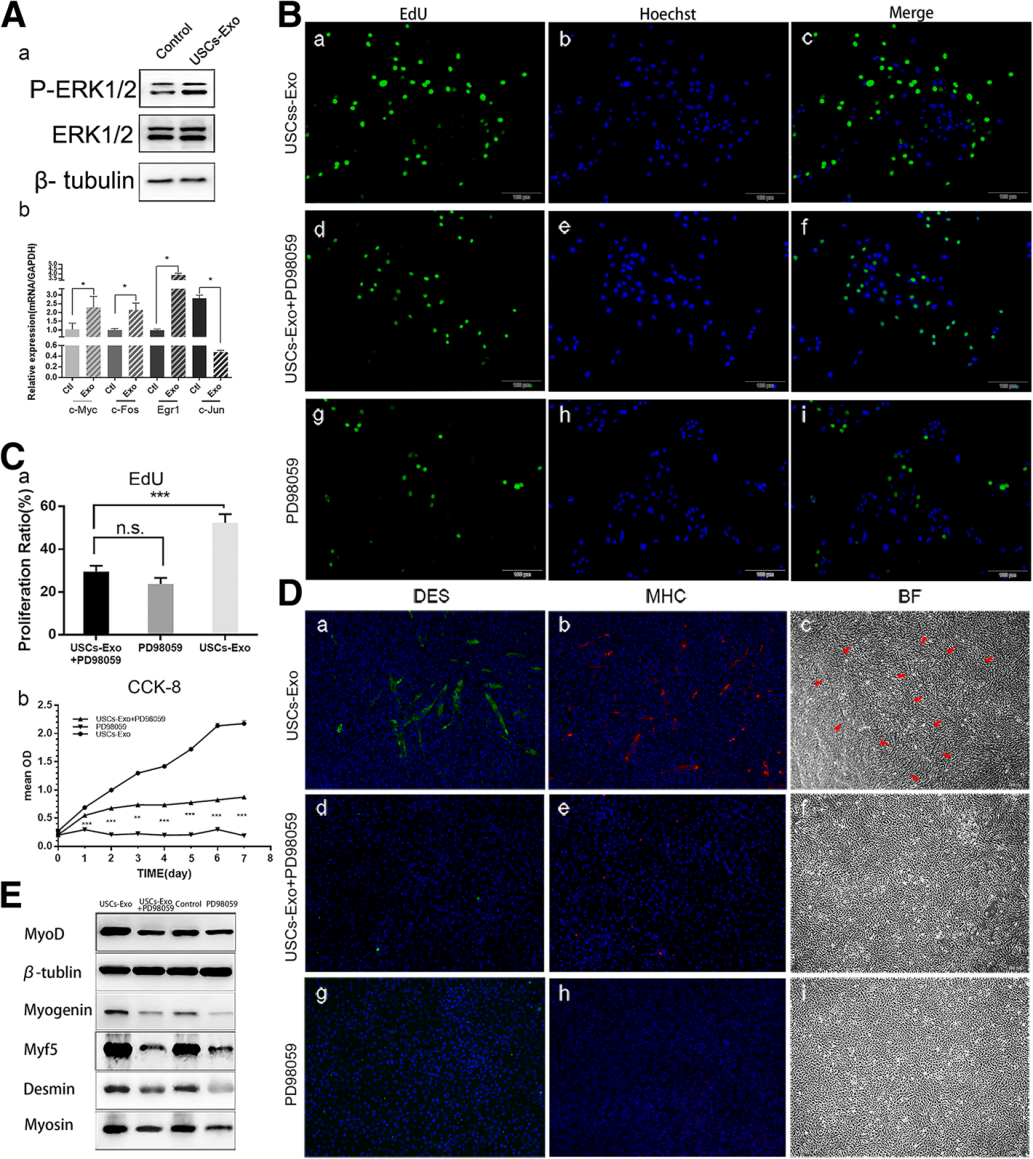
The inhibition of ERK blocked the proliferation and proliferation of SCs. **A-a** Phosphorylation of ERK was promoted in the USCs-Exo group. **A-b** Expressions of mRNAs downstream the ERK pathway were promoted in the USCs-Exo group. **B** After 24 h of using ERK inhibitor, PD98059, the percentage of proliferative SCs decreased obviously, as indicated by the EdU test. **C-a** Proliferation ratio of SCs in the EdU test. **C-b** CCK-8 test showed that PD98059 inhibited the proliferation of SCs. **D** After 24 h of using PD98059, no myotubes were observed in the PD98059 or USCs-Exo + PD98059 group compared with the USCs-Exo group (**D-a**, **D-b**, **D-c**). DES was stained green and MHC was stained red. Nuclei were stained blue. Myotubes were observed as stained DES, MHC, and arrowheads in bright field (BF). **E** Western blot analysis detected the expression of myogenic proteins in SCs under different cultivation conditions (× 100 magnification)

Previous studies revealed that Ras-ERK signaling pathway had a critical role in the proliferation and differentiation of SCs [6]. The proteins and mRNAs related to ERK signaling pathway were evaluated using Western blot analysis and RT-qPCR to investigate the underlying mechanism. The ERK phosphorylation was found promoted in SCs stimulated by USCs-Exo (Fig. 6A-a). Therefore, downstream mRNAs of Ras-ERK signaling pathway associated with cell proliferation such as c-myc, c-Fos, and Egr1 were detected in this study. Their expression was increased, and the expression of c-Jun decreased, which was related to cell apoptosis (Fig. 6A-b). These results were consistent with the promoted phosphorylation ERK1/2.

### Inhibition of ERK phosphorylation blocked the effects of USCs-Exo

ERK inhibitor (PD98059) was added into the culture medium to validate the phosphorylation of ERK. The proliferation of SCs was evaluated by EdU (Fig. 6B). Obviously, the activity of proliferation was significantly inhibited by ERK inhibitor. Compared with the USCs-Exo group (Fig. 6B-c), the effect of USCs-Exo on cell proliferation activity almost disappeared in the presence of ERK inhibitor (Fig. 6B-f, C-a). The CCK-8 test showed that the proliferation was greatly inhibited when ERK inhibitor was added into the culture medium (Fig. 6C-b). The difference in optical density (OD) between the PD98059 and USCs-Exo + PD98059 groups on day 1 was considered due to the fact that the initial effect of USCs-Exo was not eliminated although the Ras-ERK signaling pathway was blocked later. Differentiation of SCs was evaluated with DES and MHC stained by immunofluorescence. Obviously, no myotubes were observed in the USCs-Exo + PD98059 or PD98059 group after 24-h cultivation (Fig. 6D). The changes in the expression of myogenic proteins in SCs were detected using Western blot analysis (Fig. 6E). The expression of myogenin, MyoD, Myf5, desmin, and myosin significantly increased in the USCs-Exo group compared with the control group. When the ERK inhibitor was added into the medium, the expression of myogenic proteins significantly decreased in both the USCs-Exo + PD98059 and PD98059 groups.

## Discussion

In the present study, USCs-Exo were extracted and locally injected into and around the pubococcygeus muscle of rats for curing SUI. The results showed that USCs-Exo could effectively improve urodynamic parameters and help repair the injured pubococcygeus muscle in vivo. Further studies showed that USCs-Exo could promote the phosphorylation of ERK and promote the activation, proliferation, and myotube differentiation of rat skeletal muscle SCs in vitro.

In the 1990s, Delancey proposed that urethral closure was the result of contraction of the anterior portion of the pubococcygeus muscle [30]. The impairment of the pubococcygeus muscle might be the main cause of SUI [31, 32]. Currently, the Kegel exercise is widely used in clinical practice, which improves the function of the pubococcygeus muscle and promotes the contraction of the urethra and anal sphincters. Meanwhile, stem cells have been applied in treating SUI [3]. USCs are potentially superior to other stem cells for cell transplantation therapy owing their multiplex differentiation potential and easy, noninvasive methods for obtaining them [16, 17]. However, previous studies have suggested that exosomes, as messengers in intercellular communications and part of paracrine secretion, may have an important role in stem cell therapy [4–6]. Our previous studies showed that exosomes exerted several roles in regulating angiogenesis, reducing bone loss, accelerating collagen synthesis, preventing kidney complications, and accelerating the repair of the injured tissue by activating phosphorylation of Erk1/2 and PI3K/Akt [21, 33–36]. However, USCs could also serve as sources for exosome extraction [16, 17]. Therefore, this study aimed to investigate the effects of USCs-Exo in treating SUI.

Most patients with SUI have no history of trauma or surgery but natural childbirth. Therefore, in this study, an SUI rat model was established by pubococcygeus muscle injury. The urodynamic parameters significantly improved after local injection of USCs-Exo in rats with SUI. Consistently, the result of HE, Masson, and IHC staining showed better recovery of the pubococcygeus muscle in the USCs-Exo group compared with the SUI group. Therefore, it was speculated that USCs-Exo could help in the recovery of the injured pubococcygeus muscle (skeletal muscle), thereby improving the symptoms of SUI.

Being essential to skeletal muscle regeneration [37, 38], SCs are rapidly activated upon injury and then repair the impairment. Upon activation, the expression of MyoD is significantly upregulated while Pax7 is continually expressed [39]. In the present study, 24 h after cellular quiescence, SCs were stimulated by USCs-Exo, and then, the ratio of Pax7^+^/MyoD^+^ SC was increased significantly, indicating the promoted activation of SCs. In addition, the results of RT-PCR showed that the expression of Pax3/7 and cyclin B/D1/E in SCs increased after USCs-Exo stimulation, also indicating promoted activation and proliferation [40]. Further, both the EdU and CCK-8 tests indicated that USCs-Exo were able to promote the proliferation of SCs. Enhanced myotube formation under the stimulation of USCs-Exo were also observed at high cell density, further validating the important role played by USCs-Exo in skeletal muscle regeneration.

The activation and proliferation of SCs is complicated with different signaling pathways, such as Ras-ERK and PI3K/AKT, which participate in different stages and play different roles [41, 42]. A previous study showed that the Ras-ERK signaling pathway could promote SC proliferation [6]. P-ERK1/2 and ERK1/2 from SCs stimulated by USCs-Exo were examined in this study. It was found that the phosphorylation of ERK1/2 was obviously promoted. Therefore, it was speculated that USCs-Exo activated the Ras-ERK signaling pathway. Accordingly, the expression of proliferation-related genes downstream the Ras-ERK signaling pathway was evaluated, which was as presumed.

To verify the hypothesis, both USCs-Exo and ERK inhibitor(PD98059) were applied in cultivating SCs. PD98059 is a non-ATP-competitive MEK inhibitor that specifically inhibits MEK-1-mediated MAPK activation and does not directly inhibit ERK1 or ERK2. Differences between the experimental and control groups disappeared. The cell proliferation was suppressed, and no myotube formation was observed at high cell density in the USCs-Exo + PD98059 and PD98059 groups compared with the normal cultured SCs. So, we believed that it might be the MEK-ERK signaling pathway by which USCs-Exo regulated myogenesis. Besides, USCs-Exo were also able to reduce the formation of fibrosis and enhance myosin II composition during the recovery of damaged muscles, suggesting the involvement of a series of complex mechanisms, which needs further investigation.

The present study proposed that USCs-Exo were able to promote ERK phosphorylation and were positively involved in the activation, proliferation, and differentiation of SCs. The data suggested that USCs-Exo were important components of the complex mechanism for SC behavior and preliminarily revealed how USCs-Exo improved SUI. Since the study of the biological role of USCs-Exo is still in its infancy, additional discoveries of the regulatory patterns of the Ras-ERK signaling pathway mediated by exosomes may help elucidate the overall function of USCs-Exo.

## Conclusions

In summary, the present study demonstrated the importance of USCs-Exo in myogenic SC differentiation into myoblasts and identified a novel role for USCs-Exo as a cell-free agent for correcting stress urinary incontinence.

## Additional files

Additional file 1: **Figure S1.** Isolation and characterization of SCs. (DOCX 511 kb)

Additional file 2: Methods supplement. (1) Confirmation of SUI rat model and measurement of ALPP and MBV. (2) Exosome labeling and cellular uptake. (3) Antibodies. (4) Primers. (DOCX 19 kb)

## Abbreviations

ALPP: Abdomen leak point pressure
CCK-8: Cell Counting Kit -8
CM: Conditioned medium
DES: Desmin
DMEM: Dulbecco’s modified Eagle medium
FBS: Fetal bovine serum
FCM: Flow cytometry
H&E: Hematoxylin and eosin
IHC: Immunohistochemistry
MBV: Maximum bladder volume
MHC: Myosin heavy chain
PBS: Phosphate-buffered saline
SCs: Satellite cells
SUI: Stress urinary incontinence
TEM: Transmission electron microscope
USCs: Urine-derived stem cells
USCs-Exo: Exosomes secreted by urine-derived stem cells
