# Supplementary material for: Exosomes secreted by urine-derived stem cells improve stress urinary incontinence by promoting repair of pubococcygeus muscle injury in rats
Source: Stem Cell Res Ther. 2019 Mar 8;10:80. doi: 10.1186/s13287-019-1182-4 (PMC6408860; doi:10.1186/s13287-019-1182-4)
Supplement: Supplementary file 1 — Figure S1. Isolation and characterization of SCs. (DOCX 511 kb) [file 13287_2019_1182_MOESM1_ESM.docx]

**Isolation and Characterization of SCs**

SCs were obtained by harvesting a 2 × 2 × 1 mm biopsy of skeletal muscle from the hind limb of an SD rat. The cells were dissociated from the muscle fibers using collagenase IV and trypsin-EDTA mixture (Invitrogen, USA) and washed with DMEM/Ham’s F-12 (HF-12) medium (Invitrogen, USA). The cells cultured in a mixed medium consisting of DMEM/HF-12 supplemented with 10% FBS (Gibco, USA), insulin 10 µg/mL (Sigma-Aldrich), 1% GlutaMax (Invitrogen, USA), and penicillin-streptomycin (Gibco, USA). In addition, epidermal growth factor 20 ng/mL and bFGF 10 ng/mL were added every alternate day. The cells were cultured at 37 ℃ and 5% CO2 for 10 days and purified by continuously differential adhesion. The continuous growth of the SCs was passaged at every 4-5 days [[1](#_ENREF_1)]. The cells at passage 3 were fixed with 4% paraformaldehyde at 4 ℃ for 20 min and washed three times with PBS. After treating with 0.3% triton X-100 at 4 ℃ for 10 min, the cells were incubated with 3% BSA for 30 min to block nonspecific antigens. Then, the cells were incubated with anti-PAX7 antibody (Abcam, UK)[[2](#_ENREF_2)]. The cells were washed three times with PBS and incubated with FITC-labeled goat anti-mouse antibody (1:200) for 30 min in the dark, followed by flow cytometry (Guava easyCyte™，Millipore).

SC clones were observed on days 8–10 after initial seeding. The cells were a spindle or spindle-shaped with strong refraction surrounded by muscle cells (Figure S 1A). Cells were purified after continuously differential adhesion (Figure S 1B). The SCs marker protein PAX7 was identified by flow cytometry (Figure S 1C). FCM analysis revealed 99.73% purity of the isolated primary cells.

**
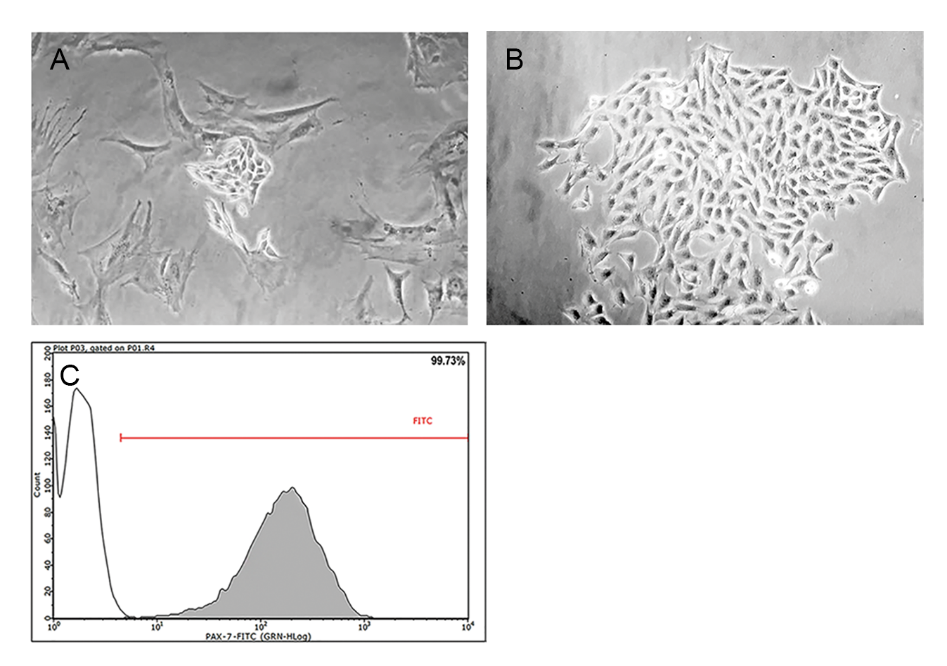
**

**Figure S 1** Characterization of SCs. **(A)** SCs (with high refraction) were isolated from skeletal muscle cells(background). **(B)** SCs were spindle shaped or spindle shaped, with high refraction**. (C)** SCs were characterized by flow cytometry using the markers PAX7.

1. Craig, J.B.; Lane, F.L.; Nistor, G.; Motakef, S.; Pham, Q.A.; Keirstead, H. Allogenic myoblast transplantation in the rat anal sphincter. *Female Pelvic Medicine & Reconstructive Surgery* **2010**, *16*, 205.

2. Danoviz, M.E.; Yablonkareuveni, Z. Skeletal muscle satellite cells: Background and methods for isolation and analysis in a primary culture system. *Methods in molecular biology* **2012**, *798*, 21-52.
