# Supplementary material for: Exosomes secreted by urine-derived stem cells improve stress urinary incontinence by promoting repair of pubococcygeus muscle injury in rats
Source: Stem Cell Res Ther. 2019 Mar 8;10:80. doi: 10.1186/s13287-019-1182-4 (PMC6408860; doi:10.1186/s13287-019-1182-4)
Supplement: Supplementary file 2 — Methods supplement. (1) Confirmation of SUI rat model and measurement of ALPP and MBV. (2) Exosome labeling and cellular uptake. (3) Antibodies. (4) Primers. (DOCX 19 kb) [file 13287_2019_1182_MOESM2_ESM.docx]

**Confirmation of SUI rat model and measurement of ALPP and MBV**

After injury, the rat urethral orifice was disinfected with iodophor, and the epidural catheter coated with paraffin oil was inserted into the bladder about 2 to 3 cm through the urethra. The experimenter applied a finger to the upper edge of the pubic symphysis of the rat to prevent the catheter from penetrating the bladder wall. After evacuating the rat bladder, the syringe was connected to the epidural catheter, and the bladder was injected with a methylene blue sterile saline solution 10 ml/h through a micro pump (Zhejiang University Medical Instrument, China). The maximum bladder volume was recorded when the blue liquid overflowed from the urethra. After emptying the bladder again, the syringe was disconnected with the catheter and the bladder was injected with sterile saline to half of the maximum bladder volume. A piece of rat whisker was cut and inserted into the nostril of the rat, causing sneeze reflex. Blue liquid leaked out from the urethral orifice indicated a positive sneezing test.

For each SUI rat, the bladder catheterization was performed before the ALPP test. The Malmgron method was taken as a reference. The rats were anesthetized with 10% urethane (1 g/kg), and placed in the supine position and a median incision was taken. The bladder was exposed, an apex of the bladder dome was made with the diameter of 1.5 mm. An epidural catheter was implanted into the bladder with a length 0.5 cm to 1 cm. Then the catheter was fixed by a purse-string suture with a 5-0 suture. The 37 °C methylene blue saline was injected into bladder with a micropump. The intravesical pressure was measured by a pressure transducer.

The abdominal leak point pressure and maximum bladder volume were measured by an animal experiment urodynamic tester (AD Instruments, Castle Hill, NSW, Australia). POWER LAB-Chart v5.2.1 urodynamics detection software was used to detect ALPP and MVB.

The bladder was evacuated by the placed epidural catheter. Then methylene blue saline was injected into the bladder, 10 ml/h. When the first drop of urine appeared at the outer urethra, the internal pressure was recorded as the abdominal leak point pressure and stop injection. At the same time, the maximum bladder volume was obtained by multiplying the micropump injection speed by the time. When the bladder is empty, continue to inject and record pressure. The measurements were repeated for several times.

**Exosome labeling and cellular uptake**

USCs-Exo were labeled with a membrane labeling dye (3,3-Dioctadecyloxacarbocyanine perchlorate, DiO) in serum-free DMEM according to the manufacturer's protocol (Absin, China) and collected as aforementioned. The labeled exosome suspension was filtered with PD Mini Trap G-10 columns (GE Healthcare Life Sciences, Pittsburgh, PA, USA). SCs were incubated at 37 °C with labeled USCs-Exo (1×1010 particles /mL) for 24 h, and then observed with a fluorescence microscope (DMI 6000, Leica Micro systems, Buffalo Grove, IL, USA).

**Antibodies**

CD29-PE(1:150) (Becton Dickinson, USA); CD73-PE(1:150) (Becton Dickinson, USA); CD90-PE(1:150) (Becton Dickinson, USA); CD44-FITC(1:150) (Becton Dickinson, USA); CD45-FITC(1:150) (Becton Dickinson, USA); CD34-APC(1:150) (Becton Dickinson, USA); HLA-DR-PE(1:150) (Becton Dickinson, USA); CD9 (1:500) (Abcam, UK); CD63 (1:1000) (Abcam, UK); CD81 (1:1000) (Abcam, UK); TSG101(1:1000) (santa cruz, USA); Pax7(1:1000) (Abcam, UK); P-ERK1/2(CST,USA); EKR1/2(CST,USA); Myogenin(1:1000) (Abcam, UK); Myosin (1:1000 for WB, 1:200 for IF) ( R&D Systems, USA); Desmin(1:10000 for WB, 1:50 for IF) (Abcam, UK); Myf5(1:10000) (Abcam, UK), β-tublin(1:5000) (Abcam, UK); Goat anti-rabbit IgG-FITC secondary antibody(1:500) (Santa Cruz); Goat anti-rabbit IgG-PE secondary antibody(1:500) (Santa Cruz); Goat anti-rabbit IgG-HRP secondary antibody (1:5000) (Santa Cruz).

**Primers**

The primers were designed by Qiagen as follows:

| gene | forward (5'-3') | reverse (5'-3') |
| --- | --- | --- |
| GAPDH | TCACCACCATGGAGAAGGC | GCTAAGCAGTTGGTGGTGCA |
| Pax3 | CAGCATCGATGGCATCCTAAGT | TTCTCTCGAAAGCGCGCTC |
| Pax7 | TGACAGCAGCTCTGCCTACGGA | GGTTGCTGAGAATGCTCATCACCT |
| cyclin A | GAGCATCTGCTGCTGAAAGTCCTG | GGTCAGCTTCCAGAAGGCTCAGT |
| cyclin B | GGAGATGTACCCTCCAGAGATCGGT | TACGGAGGAAGTGCAGAGGCAGAG |
| cyclin D1 | CGCACTTTCTTTCCAGAGTCA | AAGGGCTTCAATCTGTTCCTG |
| cyclin E | GAGTTCCCATGGAAGACTCCCA | TGCTCTGCTTCTTACTGCTGTGTG |
| Myc | CCTGAGCCCCTAGTGCTGCATGAA | TTGCTGTGGCCTCTTGATGGGG |
| Fos | AGACCAGAGCGCCCCATCCTTA | CCCTTCGGATTCTCCGTTTCTC |
| EGR1 | AAGCCCTTCCAGTGTCGAA | ATTTTGGTATGCCTCTTGCGTTC |
| Jun | GAGCTGGAGCGCCTGATCAT | ATGCAGTTCAGCTAGGGCGC |
